# Outdoor workers’ perceptions of skin cancer risk and attitudes to sun‐protective measures: A qualitative study

**DOI:** 10.1002/1348-9585.12083

**Published:** 2019-09-02

**Authors:** Marc Rocholl, Michaela Ludewig, Swen Malte John, Eva Maria Bitzer, Annika Wilke

**Affiliations:** ^1^ Department of Dermatology, Environmental Medicine and Health Theory Institute for Health Research and Education University of Osnabrück Osnabrück Germany; ^2^ Institute for Interdisciplinary Dermatological Prevention and Rehabilitation at the University of Osnabrück Osnabrück Germany; ^3^ Public Health and Health Education University of Education Freiburg Freiburg im Breisgau Germany

**Keywords:** attitude, Germany, occupational health, qualitative research, sunscreening agents

## Abstract

**Objectives:**

Since January 2015, squamous cell carcinoma or multiple actinic keratosis of the skin caused by natural ultraviolet irradiation (UVR) is recognized as occupational disease in Germany. Interventions which improve the sun protection behavior of outdoor workers are urgently needed. When developing preventive interventions, the attitudes of target groups need to be taken into consideration. Therefore, outdoor workers’ perceptions and attitudes were investigated.

**Methods:**

Seven guided, problem‐centered qualitative interviews with healthy male outdoor workers were conducted. A qualitative content analysis was used to analyze the data.

**Results:**

We found an underestimation of the perceived skin cancer risk in the seven outdoor workers and heterogeneous attitudes toward the usage of sun‐protective measures. Participants stated that the feasibility of technical sun‐protective measures depends on the size of the working area. While using a headgear seemed common, none of the participants stated using additional neck protection. Wearing long‐sleeved shirts and long trousers were considered problematic. The interviews revealed important requirements for sun‐protective clothes, especially in terms of different materials. Although the usage of sunscreen was common, our interviewees seemed to apply it wrongly.

**Conclusion:**

Risk perceptions of outdoor workers and their attitudes toward sun protection measures may influence the factual UV protection behavior in the workplace. Structures to facilitate the implementation of technical and organizational sun‐protective measures seem to be necessary. Educational interventions and clear instructions which are tailored to the individual needs and attitudes of outdoor workers are required to improve the UV protection behavior and to avoid common mistakes.

## 1 INTRODUCTION

The increasing number of nonmelanoma skin cancer (NMSC) and melanoma makes skin cancer an important public health issue.^1^ NMSC is by far the most common type of cancer worldwide.^1^ Annually, there are between 2 and 3 million new cases of NMSC on a global scale.^2^ The most important external risk factor for developing NMSC is exposure to ultraviolet radiation (UVR).^2^, ^3^ Already in 1992, the International Agency for Research on Cancer classified artificial and natural UV radiation as carcinogenic to humans (Group 1).^4^

There are about 14.5 million outdoor workers in Europe.^5^ In Germany, between 2 and 3 million employees work outdoors for a major part of their working hours—up to 75% of their working time.^5^, ^6^, ^7^ Due to their profession, outdoor workers are at increased risk for developing basal cell carcinoma and squamous cell carcinoma compared to indoor workers or the general population.^8^, ^9^, ^10^, ^11^ Since January 2015, “*squamous cell carcinoma or multiple actinic keratosis of the skin caused by natural UV irradiation*” is recognized as occupational disease no. 5103 by the German Social Accident Insurance.^6^, ^7^, ^12^, ^13^ The recognition of NMSC as occupational disease sets a strong incentive for the German Statutory Social Accident Insurance bodies for effective strategies to prevent NMSC geared at reducing UVR exposure.^14^, ^15^

Following the recommendations of the International Commission on Non‐Ionizing Radiation Protection,^16^ reducing UVR exposure by using technical and organizational sun‐protective measures, such as adjusting outdoor working hours or seeking shade whenever possible, are the most important measures in order to prevent NMSC. Unfortunately, these measures are of little acceptance and often impractical, and, therefore, do not achieve an appropriate reduction of the UVR exposure. While this requires appropriate use of personal sun‐protective measures (eg, wearing long‐sleeved shirts and trousers, wearing a hat with additional neck protection, and applying sunscreen), various studies suggest, however, as with the technical and organizational measures, the sun protection behavior of outdoor workers does not comply with these recommendations.^17^

Therefore, the development of effective, target group‐oriented interventions to encourage sun‐protection behavior in the workplace of outdoor workers is of particular relevance.^18^ When developing health‐related interventions to initiate behavior change, the attitudes and beliefs of target groups as well as perceived barriers (for instance, lack of time to apply sunscreen) need to be taken into consideration.^19^, ^20^ This is of utmost importance since these may have a major influence on the health‐related behavior and behavioral change, respectively.^21^ In this regard, the perceived risk awareness must also be taken into account as an influencing factor.^21^, ^22^ Yet, these aspects are difficult to address with quantitative approaches. Qualitative research methods serve to generate a deeper understanding of complex psychological or social issues and, therefore, present an adequate choice in this context.^23^, ^24^ Studies delivering an in‐depth insight into the multifaceted aspects of UVR protection behavior of outdoor workers are rare: Zink et al^25^ for example explored knowledge about NMSC and attitudes toward sun‐protective measures in a sample of Bavarian farmers via semi‐structured interviews.

Our study thus aims to investigate the perceived skin cancer risk and attitudes to sun‐protective measures of outdoor workers in Germany. In this paper, we will report the results of guided, problem‐centered qualitative interviews with outdoor workers.

## 2 MATERIALS AND METHODS

We conducted guided, problem‐centered interviews.^26^ Inclusion criteria were: mostly working outdoors, especially during spring and summer, and aged ≥18. Workers with previously diagnosed skin cancer were excluded. Study participants were recruited in March 2015 using both convenience and purposive sampling approaches such as recruitment letters or information sheets. Direct recruitment of potential participants was performed in the workplace, for example on construction sites. In addition, we asked study participants to identify further participants who met the inclusion criteria (snowball sampling).

All interviewees received written and verbal information about the study objectives prior to the interview. They all gave written informed consent for study participation. Each participant was informed that withdrawing approval at any time would not lead to any personal disadvantages. The principal researcher conducted all interviews face to face. The interviews were carried out in March and April 2015 at the place and time most suitable for each participant. Four interviews were held at the *Institute for Interdisciplinary Dermatological Prevention and Rehabilitation* (*iDerm*) in Osnabrück, Germany. Two interviews took place at the participants’ home and one was carried out at the participants’ workplace. A pretest was conducted with one 26‐year‐old male gardener who had been working outdoors for 8 years, to test the interview guide, for instance in view of clarity and comprehensibility of the questions. The results of the pretest were discussed with the second and the last author to modify and improve the interview guide, and the usage of open‐ended questions aimed at obtaining answers rich in information.

All interviews started with an opening trigger question concerning a typical day at work (Table 1). After that, the following topics were covered: risk perception toward occupational skin cancer, technical, and organizational sun‐protective measures, and personal protective equipment. If necessary, the order of the topics was modified according to the individual course of the interview. In addition, several materials (eg, pictures) were used to stimulate discussion of relevant topics. After each interview, the interviewer wrote a short report in which the atmosphere and special incidents were documented. The interviews lasted between 21 and 39 minutes (mean duration = 27 minutes).

**Table 1:** Topics and subtopics of the interview and exemplary questions

Introduction (Opening trigger questions):
Eg, Please, describe a typical day at work.
Topic 1: Risk perception towards occupational skin cancer
Eg, Do you perceive skin cancer caused by your profession as a problem?
Topic 2: Technical and organizational sun‐protective measures
Eg, What do you think about technical sun‐protective measures such as sun sails or parasols?
Eg, How do you assess the feasibility of the organizational sun‐protective measures, such as shifting work hours?
Topic 3: Personal protective equipment
Eg, How do you assess the feasibility of personal sun‐protective equipment like hat, shirt or sunscreen? Are there any difficulties with the application of these measures?

All audio‐recorded interviews were transcribed verbatim using the software *f4transkript* (*version 5.2, dr dresing & pehl GmbH*). We performed a quality control soon after the transcriptions by listening to each interview and comparing the transcriptions to the audio recording. All personal data were pseudonymized. We analyzed the transcripts by applying a qualitative content analysis using the software MAXQDA 11 (Version 11.1.0, VERBI Software‐Consult‐Sozialforschung GmbH). A mixed approach, consisting of inductive category development and deductive category application, was chosen. We developed deductive, theoretically derived categories based on our guiding questions. In addition, as outlined by Mayring,^27^ systematic reduction processes (eg, summarizing or context analysis) were used to derive inductive categories from the data.^27^, ^28^

## 3 RESULTS

### 3.1 Participant characteristics

Seven healthy male outdoor workers gave their consent for taking part in the study. Table 2 summarizes the sociodemographic data. Prior to the interview, no participant had received any specific educational training on sun protection.

**Table 2:** Characteristics of the study participants

Participants (n = 7)	
Mean age (range)	39.0 y (20‐58)
Mean years working outdoor (range)	21.6 y (4‐45)
Mean hours working outdoor per week during summer (range)	44.7 h (28‐60)

Profession	
Construction worker	2
Farmer	1
Gardening and landscaping	4

### 3.2 Risk perception of occupational skin cancer

The analysis of the interviews reveals that the participants perceived a certain risk of developing skin cancer. However, an underestimation of this risk perception could be derived from all statements. Especially two younger participants (interviewee 2, age: 25 years (I‐2, 25) and interviewee 5, age: 22 years (I‐5, 22)) as well as the farmer (I‐6, 58) pointed out a lower risk perception compared to other participants (‘*But a higher risk of skin cancer? I’ve honestly never thought about that before*’^1^ (I‐2, 25)). The older workers (>40 years old) stated their long work experience as well as their higher age as reasons for this awareness. The older participants also expressed that skin cancer risk or sun protection measures were not an issue at the beginning of their vocational training:

In the past ten, fifteen years, yes. As such I got aware of that and I make sure that my skin is covered, at least in direct sunlight. But only for the past ten, fifteen years, you know when you’re younger, I never took it seriously. (I‐4, 46)

Another aspect mentioned by several participants, which hindered sun‐protective behavior was the underestimated intensity of the UV radiation in Germany. From their point of view, sun protection is not as important in Germany as in Mediterranean countries:

Well, I don’t know if here in central Europe, if it is necessarily a topic, or if it is rather the case in southern countries like Spain or Italy. (I‐6, 58)

Furthermore, some participants stated that their skin gets accustomed to sun exposure (‘I’m outside every day and my skin, you know, how shall I put it, that my skin got used to it? Well, I mean, there is a certain basic tan everywhere, so to say. Because I’m outside all the time, you know’ (I‐6, 58)). Therefore, they feel no need to intensively engage in sun protection behavior.

### 3.3 Technical and organizational sun‐protective measures

The participants reported different experiences using shade to protect from solar radiation. In case of limited workspace some participants reported that using shade through deploying parasols or sun sails is a good opportunity to reduce UVR exposure during working hours:

If we pave a smaller area of four or eight square metres, where you can easily rearrange the parasol and where you spend plenty of time at the same workplace, it seems feasible. (I‐2, 25)

In contrast, using shade at wide‐ranging working areas seems unrealistic to the participants, especially when working with large machines or construction cranes:

Well, we always work with large construction equipment and, yes, sometimes there is no space. We need workspace and if, let’s say, if there is a sun sail in the way, that might go down badly. (I‐5, 22)

One participant suggested seeking natural shade in the workplace (eg, from trees) as one opportunity to counteract this difficulty:

What you can do, what I also did myself, is that I arranged the construction site so that my working process followed the natural shade. Yes, that works. (I‐3, 56)

Considering organizational sun‐protective measures (eg, shifting working hours) almost all participants expressed doubts in terms of practicability. For instance, it was stated that it was unrealistic to reschedule the main working hours in such a way as to avoid the times lot between 11 AM and 3 PM. This applies in particular if work processes were dependent on external suppliers or customers. Staying outside and being UVR exposed at midday is described as implicit part of working in an outdoor profession, especially if the individual working day can hardly be influenced by the worker himself:

Well, I don’t know how we should do that. When they say the concrete will arrive at 11am and then it takes some time, it depends, three or four hours, we can’t just say: Okay guys, the sun reached its highest point in the sky. That will never do. (I‐7, 20)

In contrast, one participant who was working as a farmer, expressed that shifting working hours is easy to implement. A key to success in this case seems to be the possibility to structure the working day on one's own.

### 3.4 Personal sun‐protective equipment

#### 3.4.1 Headgear

All participants reported that if helmets are not mandatory they would use a normal baseball cap when working outdoors, but no one expressed to have additional neck protection. Broad brimmed hats or caps with neck protection were seen as a good opportunity to protect areas which are intensively exposed to the sun such as neck or ears. On the other hand, the design of the headgear which is unfamiliar to the participants, seems to be a factor influencing their use (‘*It's kind of unusual to me. I don't go for it, I don't know, that's just my attitude’* (I‐4, 46)). In addition, especially the construction workers expressed doubts with regard to occupational safety regulations, for instance in terms of a restricted visual field caused by broad‐brimmed headgear or the necessity to combine headgear with hearing protection:

Well if I put it like that, I think that our vision would be impaired. It would be difficult to recognise the loads which are coming down, you know, to see them. (I‐1, 46)

Based on our findings, it can be noted that study participants seem to prefer light and breathable headgears in bright colors. All participants pointed to the importance of testing different headgears as a prerequisite for long‐term use. The results also reveal personal preferences as an important influence on the usage of different types of headgear (Table 3).

**Table 3:** Requirements concerning personal sun‐protective equipment mentioned by the interviewees

Requirements on headgear
Breathable materials (air permeable)
Good fit (eg, on the head)
High wearing comfort
Bright colors
Design should not be too prominent (eg, untypical appearance of the neck protection)
Must be compatible with other occupational safety devices (eg, hearing protection)
Neck protection does not stick to the skin when sweating

Requirements on sun‐protective clothes
Bright colors
Breathable materials (air permeable)
Should not stick to the skin when sweating
Comfortable to wear
Should be simple and feasible (eg, without any loops or slings)
Cheap and accessible for each employee
Depending on individual preferences: cotton or high‐tech synthetic microfiber
Must be compatible with other occupational safety devices (eg, regarding flammability)

Requirements on sunscreen
Fast‐absorbing
Non‐greasy
Easy to apply
Well spreadable
Financially affordable for target group

#### 3.4.2 Clothes

None of the participants indicated to work shirtless during the summer months. Some of the older participants stated that they did that in the past, especially at the beginning of their working life (‘*Once before, one has worked shirtless but that was a young age*’ (I‐1, 46)). Wearing long‐sleeved shirts and long trousers between March and October is described as additional burden, because of the experienced heat accumulation. The participants furthermore voiced excessive sweating as problematic while working:

It’s just that, at some point you start sweating. It is uneasy to feel fabric on the skin. (I‐2, 25)

Experiences with different materials, for example, cotton or high‐tech synthetic microfiber, to counteract this problem varied among the participants. In this context, preferring one or the other material seems to depend on individual preferences. Our results indicate that wearing suitable clothes to reduce UVR exposure can be positively influenced, above all if outdoor workers get the opportunity to participate in the decision‐making process. Thus, all participants demanded a field test of potentially appropriate clothes, which seems to be a key success factor for wearing sun‐protective clothes. Table 3 summarizes the requirements concerning suitable clothes as described by the interviewees. Industrial safety regulations (eg, concerning low flammability textiles in case of flying sparks) should, in addition, be taken into consideration.

#### 3.4.3 Sunscreen

Aside from one participant (‘*Honestly, I see no need to apply sunscreen*’ (I‐6, 58)), all interviewees confirm the use of sunscreen while working outdoors during summer. However, the majority of the participants did not know which sun protection factor (SPF) they were applying. Most of the interviewees reported that they used medium or high protection factors, but without specifying the exact SPF. Only one interviewee reported that he applied SPF 40 or higher because of being highly exposed to ultraviolet radiation:

Well, I use sunscreen with at least sun protection factor 40, because I am intensively exposed to the sun. I guess sunscreen with a sun protection factor of twenty‐five or fifteen isn’t sufficient. (I‐4, 46)

When asking the outdoor workers for the time of application, they said they applied the sunscreen one to three times a day, especially during their breakfast or lunch break as well as in the morning before starting their working day. One participant, on the other hand, stated to apply sunscreen only once a day:

That’s enough if I do it once in the morning, because it lasts for the whole day. (I‐3, 56)

The interviewees furthermore indicated that applying sunscreen depends on the weather conditions of the respective day. The use of sunscreen is not considered necessary, if UVR exposure is estimated to be low, for example when it is cloudy or the temperatures are low (eg, under 20 degrees). The interviewees described various problems when using sunscreen during working hours: slowly absorbing sunscreens, greasy and sticky consistency of products which leave a (white) residue on the skin as well as greasy hands after application. Forgetting sunscreen application was another problem highlighted by the participants. Some interviewees pinpointed in addition that there were only few possibilities to (re‐)apply the sunscreen during the working day because of time pressure as a result of tightly tapped workflows:

When the working day is very stressful, you sometimes forget to reapply sunscreen. (I‐7, 20)

We found diversified preferences with regard to the type of sunscreen: some participants preferred spray, others preferred creams or gels.

To sum up, the usage of sunscreen was common among the interviewees although we assume that the sunscreen is often applied incorrectly, including in terms of the frequency of sunscreen use or the time of application. This results, most likely in insufficient protection against UVR. The requirements on sunscreens as derived from our interviews are summarized in Table 3.

## 4 DISCUSSION

The aim of our study was to investigate the perceived skin cancer risk and attitudes toward sun‐protective measures of outdoor workers in Germany by carrying out guided, problem‐centered qualitative interviews.

Our findings reveal an underestimation of outdoor workers’ risk perception of getting skin cancer. A previous study with Bavarian farmers showed heterogeneous results regarding the perceived risk of developing skin cancer compared to the general population.^25^ In a sample of outdoor workers, Zink et al^17^ found that a low risk perception of skin cancer is associated with an insufficient use of protective measures. However, only few studies focus on the association between risk perception and sun‐protective behavior, with inconsistent results.^29^ Malenga^30^ could not show an association between perceived susceptibility of getting skin cancer and engagement in sun protection behavior in dairy farmers. While Hammond et al^31^ pointed out an association of high‐risk perception and increased usage of sun protection measures, Grandahl et al^32^ could not confirm these results in a sample of Danish outdoor workers. Renner et al^33^ examined the influence of age on risk perception and found that risk awareness rises with increasing age and declining general health.^33^ On the other hand, Nahar et al^29^ found that higher age and the number of working years in an outdoor profession are associated with better sun protection behavior compared to younger workers, which was confirmed by further studies.^29^ This was corroborated by the findings of Zink et al,^25^ who found low interest in sun protection among especially young male farmers in Germany. Considering the fact that NMSC is associated with cumulated occupational exposure to UVR, sustainable primary prevention concepts should occur as early as possible, for example, during vocational training.^34^

According to Schwarzer,^21^ risk perception is an important but not the only predictor for behavioral change. In addition, the perceived positive outcome expectancies as well as the self‐efficacy are further important cognitions in behavioral change processes.^21^, ^22^ It can be assumed that outdoor workers’ sun protection behavior can hardly be influenced by isolated risk communication (eg, fear appeals) because risk perception does not have to lead to the necessary resources that are needed to initiate behavioral change. Therefore, sun protection interventions should contain appropriate risk communication coupled with resource communication (eg, to improve self‐efficacy) to induce behavioral change.^21^, ^25^ Furthermore, disease‐related knowledge seems to be an important aspect. However, it should be noted that isolated knowledge transfer in an intervention is not a predictor of behavioral changes.^17^, ^25^, ^29^, ^31^

In addition to perceived skin cancer risk and knowledge of skin cancer, the most important factor to improve sun protection behavior of outdoor workers is the perceived social support in the workplace.^29^, ^31^, ^35^ Hence, besides training and counseling of workers, a key factor of preventing occupational skin cancer seems to be promoting social support in the workplace. Ruppert et al^34^ found that despite legal obligations, half of a sample of German apprentices from different outdoor professions are not provided with sun‐protective measures from their employer. Thus, mandatory sun‐safety polices should be enhanced.^34^, ^35^ This aspect is particularly important in view of the high costs of sun‐protective measures^36^ and rather low income of outdoor workers.

According to national and international occupational safety and health regulations,^37^, ^38^ technical and organizational measures should be checked for the protection of employees as a matter of priority. Concerning the feasibility of technical and organizational sun‐protective measures (eg, providing shade), the participants did not identify many implementation opportunities in their daily work. These findings are in line with other studies.^39^, ^40^ The majority of the participants also expressed doubts on practicability. However, one of them described an approach (arrangement of construction sites to use natural shade) that seems to be feasible at least in certain work contexts. To what extent these measures can be realized is not so much a question of behavioral prevention, but rather a question in the field of structural prevention.^41^ It appears that implementing technical or organizational measures strongly depends on the individual workplace (eg, the possibility to organize the working day autonomously).

Against this background, personal sun‐protective measures are important. We have found a high acceptance of using some kind of headgear to reduce UVR exposure among our outdoor workers. This might be due to an overall common usage of headgear among male outdoor workers.^42^, ^43^ In accordance with findings from a study with Austrian tinsmiths,^44^ the usage of protective headgear—such as caps with neck protection—depends on the design of these products. Designs which are considered by outdoor workers as too obtrusive (eg, untypical appearance of the neck protection) are less accepted. Furthermore, in the selection of a suitable sun‐protective headgear, the involvement of an occupational health and safety expert might be necessary in order to take health and safety regulations into account, for instance with regard to the restriction of the visual field.

Our results indicate that the usage of sunscreen is often incorrect, especially regarding the time and frequency of application, which may lead to a reduced protective effect.^45^, ^46^ Previous research also found a lack of appropriate sunscreen usage, with regard to the improper amount applied on the skin—even if study participants had received individual training.^47^ Also, the present study illustrates the participants’ requirements on sunscreen formulations. Although the statements regarding the different sunscreen types (spray or milk and gel formulations) varied among the participants, the requirements (eg, fast‐absorbing, well spreadable; Table 3) are in accordance with previous studies.^42^, ^44^, ^47^ In order to improve the usage of sunscreens, to reduce application errors and to address common barriers, such as forgetting to apply sunscreen, educational concepts need to be developed.^41^, ^47^, ^48^ These could include UV photography to show individual application gaps with a view to improve the individual *sunscreen application technique*.^49^

This study has some strengths and limitations. Although we have used different recruitment strategies, we were unable to recruit female outdoor workers. This may have influenced our results even though men are overrepresented in outdoor professions (eg, construction sector, agriculture and forestry, and fishing industry).^50^ Women might have different perceptions of skin cancer and also different attitudes toward personal sun‐protective measures than men.^43^, ^51^ As an example, Kearney et al^43^ pointed out that there are differences between women and men regarding the selection of sun protection measures. In addition, studies showed increased awareness among female farmers.^25^ For this reason, it is important that future studies also take female outdoor workers into account. Despite the fact that qualitative research is not at all aimed at representative sample sizes,^52^ our sample size was rather small. However, the data from the interviews are rich in information, particularly with regard to the diverse perceptions of the interviewees and significantly contribute to the current discussion. In this context, we were able to identify several new aspects, for instance a more comprehensive understanding of the reasons why outdoor workers do not comply with actual recommendations in terms of UVR protection. These insights are important when developing sun protection interventions.^19^, ^20^, ^53^ Further investigations could also take into account that tanned skin is considered as attractive among wide parts of the general population^54^, ^55^ and selected occupational groups, for example, lifeguards.^56^ This may also be an important aspect for outdoor workers to refuse sun‐protective measures.

## 5 CONCLUSIONS

To our knowledge, this is the first time that a study delivers deeper insights into the attitudes and perceptions of different occupational groups of German outdoor workers via a qualitative research approach. That seems important, because risk perceptions of outdoor workers and their attitudes to sun protection measures are likely to influence the UVR protection behavior in the workplace. We found a rather low‐risk awareness with regard to skin cancer and some critical and heterogeneous attitudes toward various personal UVR protection measures. Educational interventions and clear instructions are required, tailored to the individual needs and attitudes of outdoor workers. Aside from individual behavior‐based approaches to prevent NMSC, it seems necessary to create structures to facilitate the implementation of technical and organizational sun‐protective measures, including by means of occupational safety regulations. Ideally, the correct use of sun‐protective measures should become a natural part of the professional activity already during vocational training.

## DISCLOSURE

*Approval of the research protocol:* N/A. *Informed consent:* All study participants gave written informed consent for participation. *Registry and registration no. of the study/trial:* N/A. *Animal studies:* N/A. *Conflict of interests:* Authors declare no conflict of interests for this article.

## AUTHOR CONTRIBUTIONS

MR collected, analyzed, and interpreted the data; ML supported analysis process; MR developed the interview‐guide; ML and AW reviewed the interview guide; participants were recruited by MR and ML; E.‐MB and MR conceived the idea for this study; E.‐MB and SMJ supervised the project; all authors contributed to the final manuscript.

## ENDNOTE

1 The interviews were conducted in German. The transcription and analysis were also carried out in German. The quotations which are relevant for this manuscript, have been translated by the first author for the purpose of this publication.
